# Dynamics of volcanic vortex rings

**DOI:** 10.1038/s41598-022-26435-0

**Published:** 2023-02-09

**Authors:** Fabio Pulvirenti, Simona Scollo, Carmelo Ferlito, Florian M. Schwandner

**Affiliations:** 1 School of Surveying and Land Information Engineering, Henan Polytechnic University, Jiaozuo, 454000 China; 2 Istituto Nazionale di Geofisica e Vulcanologia, Osservatorio Etneo, Catania, Italy; 3 Dipartimento di Scienze Geologiche, Università di Catania, Catania, Italy; 4 NASA Ames Research Center, Moffett Field, CA USA

**Keywords:** Volcanology, Applied physics

## Abstract

Vortex rings can easily be generated in the laboratory or with homemade devices, but they have also been observed on volcanoes, since the eighteenth century. However, the physical conditions under which volcanic vortex rings form are still unknown. In order to better understand this phenomenon and provide clues on the dynamics of the volcanic vortex rings, we performed a series of finite element simulations to investigate which model configuration leads to the rings formation that best matches the field observations. Results show that the formation of volcanic vortex rings requires a combination of fast gas release from gas bubbles (slugs) at the top of the magma conduit and regularity in the shape of the emitting vent. Our findings offer important insights into the geometry of the uppermost portion of vortex-forming volcanic conduits. Volcanic vortex ring studies may form the basis for a cross-disciplinary assessment of the upper conduit dynamics of volcanic vents.

## Introduction

Vortex rings (a.k.a. “smoke” rings) have been studied since the end of the nineteenth century^1–3^ but Sir William Thomson, later known as Lord Kelvin, was among the first to explain their formation mechanism^4^. Starting from the assumption of a homogeneous, incompressible, frictionless fluid and using the fundamental equations of fluid motion, he demonstrated that, in a perfect fluid, vortex filaments turn in upon themselves forming closing rings and that these vortex rings follow specific rules of rotational and translational motion. In recent times, the motion of vortex rings of small cross sections was studied by Saffmann^5^. Based on previous studies^2,6–12^, Saffmann derived a formula for the velocity of a vortex ring in an ideal fluid and extended his findings to the case of a vortex ring subject to viscous diffusion. His results were confirmed by Kaplansky^13^. First attempts to mathematically calculate the two-dimensional trajectories of an ideal vortex pair near an orifice were made by Sheffield^14^, considering different angles between the conduit (where the airstream starts) and the outside wall. Vortex rings have been also investigated through experiments^15–20^. As reported in Silver^21^, the construction of a first “smoke cannon machine” is attributed to Peter Guthrie Tait. He constructed a wooden box with a circular hole carved at one end, then he covered the other end with a tightly stretched towel and placed ammonia and sulfuric acid inside the box. He observed that vortex rings were generated by striking the towel and that if an orifice shape other than a circle was used (like an elliptical or squared hole) the rings shook and vibrated before gradually taking on a circular shape. Other studies investigated the formation of vortex rings via numerical simulations^22–30^. A summary of the history of vortex rings can be found in Meleshko et al.^31^.

**Figure 1:**
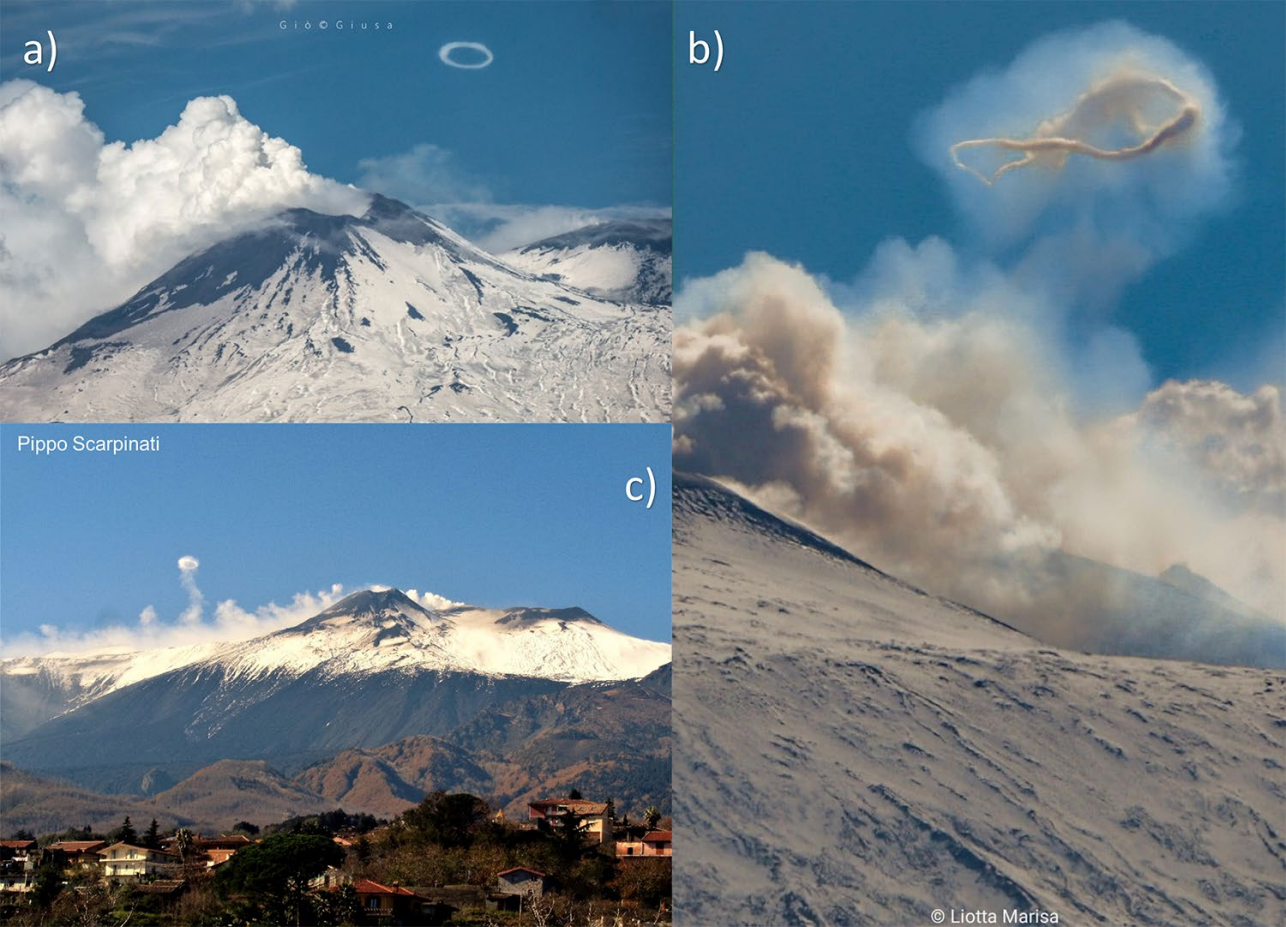
Examples of volcanic vortex rings having a radius of tens of meters observed at Etna (Italy). Photo courtesy of: (**a**) Giò Giusa; (**b**) Marisa Liotta; (**c**) Pippo Scarpinati.

Vortex rings have also been observed on volcanoes. A volcanic vortex ring will be hereafter indicated as VVR. First observations of VVRs at Etna and Vesuvius volcanoes (Italy) date back to 1724 and are documented in an engraved plate from 1755^32^. A paper on VVRs was published by Perret^33^, who observed them on Etna (Italy) in 1910. In more recent times, VVRs have also been observed at different volcanoes (e.g., Redoubt (Alaska), Tungurahua (Ecuador), Pacaya (Guatemala), Eyjafjallajökull and Hekla (Iceland), Stromboli (Italy), Aso and Sakurajima (Japan), Yasur (Vanuatu), Whakaari (New Zealand) and Momotombo (Nicaragua)) (Table 1). Photos of VVRs at Etna volcano are shown in Fig. 1. Supplementary Appendix A (Videos 1, 2), B and C include videos, articles and photos available on the internet.

**Table 1:** List of volcanoes that have produced vortex rings.

Volcano	Volcano type	Bulk-composition									
		Andesite	Basalt	Picro-Basalt	Bas-Andesite	Trachyte-Andesite	Trachyte-Basalt	Trachyte	Trachyte-Dacite	Dacite	Ryolite
Aso (Japan)	CA-SV	X	X	X							
Etna (Italy)	SV	X	X	X		X	X	X	X		
Eyjafjallajökull (Island)	SV	X	X	X		X	X	X	X		
Hekla (Island)	SV	X	X	X	X					X	X
Momotombo (Nicaragua)	CA-SV	X	X	X	X						
Pacaya (Guatemala)	COM	X	X	X	X					X	X
Redoubt (Alaska)	SV		X	X	X						
Sakurajima (Japan)	SV	X	X	X	X					X	
Stromboli (Italy)	SV	X	X	X	X	X					
Tungurahua (Ecuador)	SV	X	X		X					X	
Yasur (Vanatu)	SV	X			X						
Whakaari (New Zealand)	SV	X			X					X	

*CA* Caldera, *SV* Strato-volcano, *Com* complex.

The mechanism regulating the generation of vortex rings is well understood from laboratory experiments. These experiments (Supplementary Appendix A, Video 3) show in fact that a vortex ring can easily be generated by using a piston to push a portion of fluid out of a container through a nozzle and that the characteristics of the generated vortex ring (size, velocity, vorticity), as well as the presence of a trailing jet, depend on the piston velocity profile and on the piston stroke ratio (fluid length to nozzle diameter, L/D). In some cases, a single ring with no trailing jet will form, in other cases the ring will appear with a trailing jet and then it will pinch-off, travelling with its own self-induced velocity, following a poloidal motion. Alternatively, it can be overtaken by the trailing jet and be destroyed^24,34–36^. The authors believe that a similar mechanism exists on volcanoes, though important questions arise: what represents the role of the piston in the volcanic conduit? Why do we observe vortex rings at many but not at all volcanoes, and why sometimes a volcano can produce several rings in a day but other times none? Answering these questions is not straightforward since, in complex natural settings like volcanoes, the conduit cannot be directly examined. In order to provide answers, we combine the observed characteristics of VVRs (size, shape, color, velocity, residence time, etc.) to the knowledge of the morphology of volcanic vents and of the dynamics of the upper magma conduit system and, with the use of finite element simulations, we infer the possible VVRs formation mechanism.

## Observations

Assessing the characteristics of VVRs (e.g., temperature, velocity, vorticity) is not an easy task: they are sporadic phenomena and predicting their occurrence has been elusive at best. Even after the emitting vent has been identified, the speed of the formation process and its evolution, combined with weather conditions, can make it hard for researchers to perform direct measurements. Fortunately, several videos and pictures (most of them produced by amateur videographers or from cameras installed by researchers on volcanoes) have captured these phenomena, so we can extract information of their characteristics from these observations (see some examples in Supplementary Appendix A–C). In absence of rings, passive degassing (mostly water vapor mixed with other gases, prevalently SO_2_ and CO_2_) is usually observed at vents, visible as result of condensation in the cold atmosphere. Observations show that a few instants before the ring appears, a puff of hot water vapor is quickly emitted from a vent. The water vapor rolls-up at the vent borders forming tube vortices, while its central part is pushed out at higher speed, dragging all the vortices with it. The ring forms and becomes visible by condensation, with a radius comparable to that of the emitting vent. VVRs can be ejected from small (radii of few meters) circular vents (like the “puffers” described in Del Bello et al.^37^ but also from big craters (with radii of tens of meters, as seen at Etna in Fig. 1). From observations, we can also extract the following distinctive characteristics:

1. *Small core radius* (as defined in Sullivan et al.^28^). This indicates that the ratio between the length of the ejected fluid (L) and the diameter of the emitting vent (D) must be comparable (L/D ≤ 2). In a video from a laboratory experiment (Supplementary Appendix A—Video 3), it can be seen that the ring core radius grows proportionally to the stroke ratio L/D^38^. Assuming the slug gas to be contained in a cylinder of diameter D and height L, the volume of ejected water vapor can then be calculated as:
  1 $${\text{V}}_{{{\text{H2O}}}} = \, \pi \times \left( {{\text{D}}/{2}} \right)^{{2}} \times {\text{L}},$$
  but, as shown in Gharib et al.^34^, L is also related to the speed of the pressing source U_p_ as:
  2 $$L={\int }_{0}^{t}{U}_{p}dt,$$
  where t is the duration (in s) of the applied overpressure. For L to be comparable to D, the duration of the overpressure pulse must be small. It’s then plausible to assume that VVRs are generated by a short time pulse, such as the explosion from gas slugs during transient Strombolian eruptions.
  Following (1), and for a vent diameter typical of puffers (D = 2–3 m), an upper limit for the gas volume of the slug would be 21 m^3^ (if L/D = 1) and 42 m^3^ (if L/D = 2). Similar values have been found by Vergniolle and Brandeis^39^ by matching synthetic acoustic pressure waveforms to recorded signals from 36 eruptions at Stromboli and by Ripepe and Marchetti^40^ by infrasound measurements. VVRs with a larger core radius and a trailing jet, as the ones sometimes observed at Mount Etna, suggest instead that L/D ≥ 4^34^. This may indicate much larger gas slug volumes or longer pulse duration, but this case is not considered in this work.
2. *White color*. This indicates that VVRs mainly consist of water vapor, condensed to liquid aerosol. In few cases however, they appear brownish and this is probably due to the capture of some ash present in the atmosphere and/or in the volcanic conduit. Precise measurements of their chemical composition may be acquired by ground-based remote sensing spectroscopy during field campaigns, but unfortunately this approach has not yet been attempted.
3. *High temperature at the vent*. Thermal cameras installed on Yasur volcano (Vanuatu) show that when VVRs form, their initial temperature is close to apparent magma temperature (Supplementary Appendix B—Article 1) but as soon as the VVR moves upwards and interacts with the surrounding cold air, it cools down fast.
4. *Variable residence time*. In a video (Supplementary Appendix A—Video 1) we can observe that, while rising up, the previously undisturbed air in front of the VVR is entrained by the swirling motion (flux entrainment) while a wake is formed behind the ring. The balance between the entrained flux and the flux shed to the wake, regulates the time the ring resides in the atmosphere. Therefore, small rings, which contain only a small portion of water vapor and do not show a trailing jet, disappear in tens of seconds while large rings, with a radius of tens of meters, have been observed to persist for a few minutes and can reach a maximum height of a few kilometers above the vent^41^. However, interaction with flux streams (e.g., wind) and non-uniform dissipation processes (e.g., presence of volcanic ash) may induce instability with consequent warping and disruption^42^. Combining the information coming from observations of the maximum height and residence time (Supplementary Appendix A joined to other volcanological observations), we can infer an average ascending speed of 2–40 m/s. The upper limit (40 m/s) is in agreement with the work of Suwa et al.^41^ who studied the February 2011 Sakurajima volcano eruption combining data from observations with numerical 3D simulations.
5. *Generated from andesitic/basaltic stratovolcanoes*. This means that magma viscosity is low to moderate (SiO_2_ content between 50 and 60%), which may favor the production of high-speed slugs before the VVR is formed. The lists of VVR forming volcanoes and of VVR characteristics, as extracted from the observations, are summarized in Tables 1 and 2 respectively.

**Table 2:** Main ring characteristics (nominal values) obtained from observations.

Parameters	Range
External radius	5–100 m
Core radius	~ 0.1–1 m
Nominal speed of ascent	2–40 m/s
Residence time in air	1–10 min
Height from the vent	< 1 km
Color	white/brown
Temperature	> 700 K
Rate of emission	From rare up to several per day

## Dynamics of volcanic vortex ring formation

**Figure 2:**
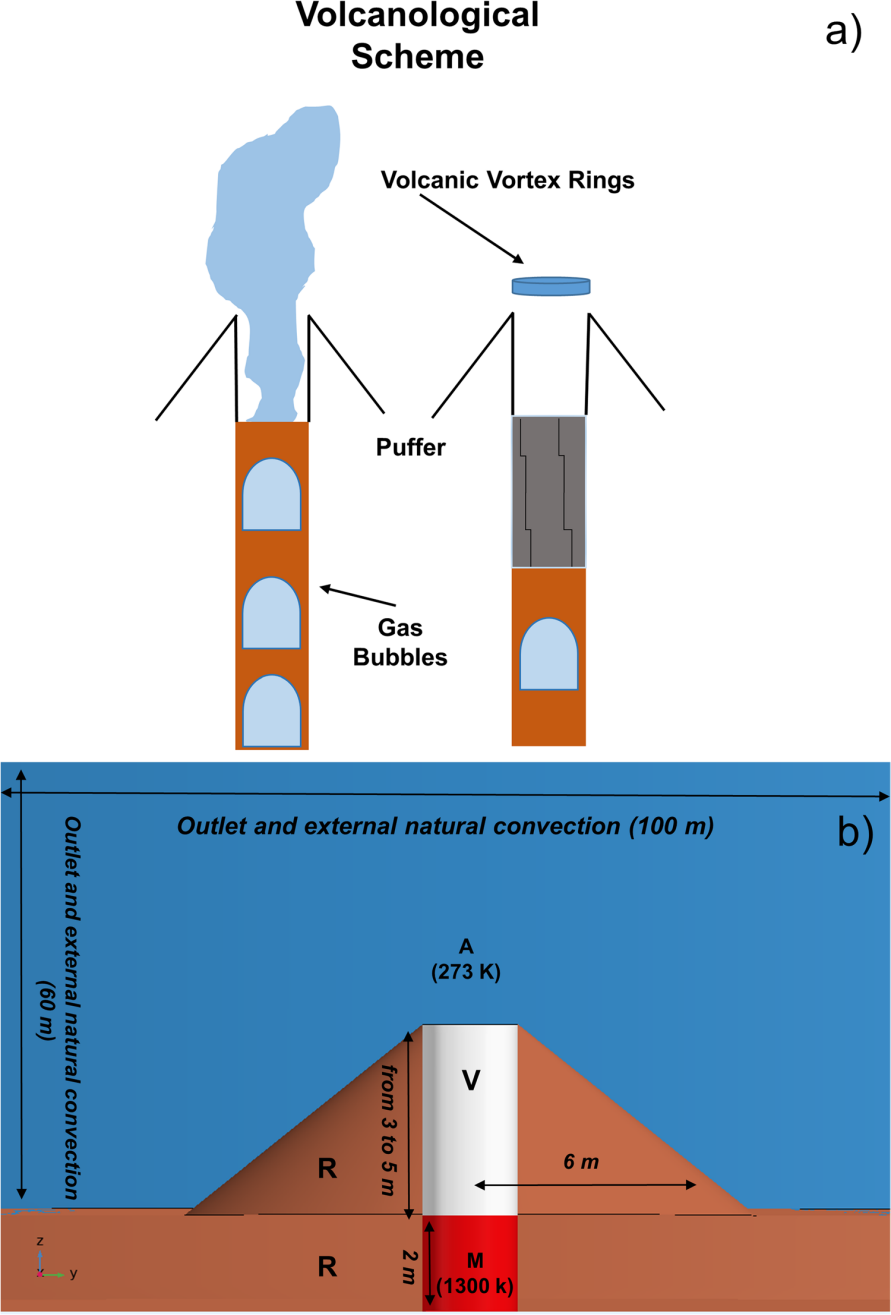
(**a**) Scheme showing the steps leading to the formation of Volcano Vortex Rings. (**b**) ZY cross section and boundary condition of the geometry used for the model. *M* magma conduit, *R* puffer and underlying rocks, *V* water vapor and *A* air.

In the magma conduit, small gas bubbles form at about 3 km depth (the exsolution level), merge by coalescence and buoyantly rise up in the form of large, pressurized gas pockets (slugs) which can potentially reach the surface, depending on the balance between gas volume and magma viscosity. Seismo-acoustic observations applied to Aso volcano (Japan) have shown that, before a Strombolian explosion, the uprising slug may reach a maximum velocity of 160 m/s^43^. However, the above speed value is probably an upper limit because the mechanism which creates Very Long Period (VLP) signals is highly debated and thus the depth of their generation is highly speculative. The depth together with the time delay of signals translates into the derived velocity. Therefore, if signal creation happens at shallower depth in the conduit, the velocity value decreases significantly. When the pressurized slug arrives in proximity to the top of magma conduit, two scenarios are most likely to occur: (1) the magma conduit’s top is open (this work). In this case, the slug experiences a pressure difference with the outside atmosphere and explodes slightly below an overlying thin magma shell. Doppler radar installed at Erebus volcano^44^ show that uprising magma shell velocities could reach a maximum value of 60 m/s and the release of the underlying gas occurs in less than half a second; (2) the top part of the magma conduit cools down and solidifies, forming a thin plug, while gas accumulates underneath it. Additional pressure generated from deeper levels (e.g., further rising of gas bubbles), or a depressurization from the top (e.g., if the plug gets partially fragmented or part of it sinks back inwards into the conduit) can further destabilize the system promoting the explosion. The slug explosion lifts the plug up, breaking it apart partially or entirely^45^. In both scenarios, part of the energy from the slug explosion is dissipated viscously or converted into infrasonic and seismic energy while another part is transferred as net kinetic motion of the hot vapor, which is pushed into the surrounding cold air. In both cases, the impulsive overpressure from an exploding slug could resemble the pushing force of the piston in laboratory experiments and is then a valid candidate as source for the generation of VVRs. The difference between both scenarios when compared to a piston is the diameter of the piston in relation to the diameter of the conduit. In scenario 1 both diameters will be the same from the beginning. In scenario 2 the piston will increase in size while the plug is breaking apart, making way for the gas to escape. Hence, VVRs could be formed during the presence of Strombolian activity consisting of a series of discrete explosions separated by intervals of seconds to several hours^46^ and this would explain the variability in the number of emitted rings. A schematic example is given in Fig. 2a.

## Results

### Numerical simulations

In order to reproduce the process of formation of VVRs, we developed a complex time-dependent non-isothermal finite element model, using the commercial software COMSOL Multiphysics (https://www.comsol.com/). The simulations focus on rings generated by a puffer, which have been observed on Stromboli volcano^19^, but we speculate that a similar mechanism occurs for large emitting vents or small craters as well. Following indications from field observations and literature^37,47–49^ we built a 2D-axi-symmetric geometry including the very top portion of a magma conduit with surrounding hosting rocks, a puffer and the external air environment. The ZY cross section of the 3D revolution, with corresponding dimensions, is shown in Fig. 2b and geometric parameters are summarized in Table 3. The model considers a system with three fluid domains: magma, water vapor as observed during volcanological observations, and air, while the solid boundaries of the puffer, the magma conduit and the hosting rocks are considered via specific boundary conditions only. Water vapor and air properties are functions of temperature, while magma properties (mafic magma) are set as constant, because of the very small portion of magma considered. Material parameters are summarized in Table 4, while used functions and solved equations are described in Supplementary Appendix D. It’s worth saying that we do not explicitly simulate the slug, neither the slug rise, but we rather incorporate the effect of the slug explosion. In particular, we assume that a slug has reached the top part of the conduit, growing in size up to a radius almost comparable to the conduit radius, and that due to the imbalance between its internal pressure and the pressure above the magma shell, it explodes. The explosion generates an overpressure that pushes the passively degassed water vapor out from the puffer to the air domain, generating the ring. We model the slug explosion by defining an inlet condition, in the form of a pulse function (Gaussian time-dependent) on a boundary located at 10 cm below the conduit top, to take into consideration the presence of a thin magma shell above. The Gaussian amplitude is parametrized to assume values of 1 kPa, 10 kPa or 100 kPa and the total duration of this pulse is 0.2 s. Since the kinematics of VVRs is mainly turbulent, and to consider all the physical processes involved, we couple the turbulent fluid flow interface to the transport of diluted species and to the heat transfer interface. Coupling these three physics allow us to accurately define the behavior of the fluids in terms of velocity, pressure, turbulent kinetic energy and specific dissipation rate and to consider convection, mass transport, thermal buoyancy and viscous dissipation. The turbulent fluid flow interface solves for the Reynolds Averaged Navier–Stokes equations (RANS) with a turbulent k-ω approach^50^. The transport of diluted species interface is used to solve for the fluid concentration and to consider diffusion and convection transport mechanisms. The heat transfer interface solves for the fluids temperature, assuming the temperature of the air domain (A) to be 273 K and a temperature of 1300 K for the magma layer (M) into the conduit. The temperature of the water vapor in the puffer (domain V) is interpolated, over the puffer height, between these two values. At the boundaries of the air domain, an *outflow* boundary condition simulates the environment’s continuity. On the same boundaries, heat flux conditions assure external natural convection and the thermal exchange with the surroundings. The Kays Crawford model^51^ is used for mass and heat transport turbulence. Finally, the conduit boundaries, the hosting rock and the puffer boundaries are considered via *wall no-slip, wall functions* and thermal insulation boundary conditions. Used boundary conditions are shown in Fig. 2b. The finite element mesh consists of triangular elements with P2 + P1 discretization (second order polynomial functions for the velocity and first order for the pressure). The model solves for a total time of 10 s and timesteps are automatically adjusted by the software, so to have a smooth increment towards the solution.

**Table 3:** Geometrical parameters.

Parameter	Value	Description
d1 [m]	1	Conduit radius
d2 [m]	1	Puffer top radius
d3 [m]	6	Puffer basal radius
d4 [m]	30	Air domain radius
h1 [m]	3 to 5	Puffer height
h2 [m]	50	Air domain height
h3 [m]	2	Magma conduit height
Press [kPa]	1 to 100	Slug overpressure

**Table 4:** Material parameters.

Parameters	Air	Water vapour	Magma (mafic)
Dynamic viscosity [Pa*s]	eta(T)	eta1(T)	1000
Density [kg/m^3^]	rho(T)	rho1(T)	2600
Thermal conductivity [W/mK]	k(T)	k1(T)	0.6
Heat capacity at constant pressure [J/(kg*K)]	Cp(T)	Cp1(T)	1450
Initial temperature T_0_ [K]	273	Interpolated	1300
Diffusion coefficient in excess of air [cm^2^/s]	N/A	1.7	N/A
Ratio of specific heats	1.4	1.1	1.45

**Figure 3:**
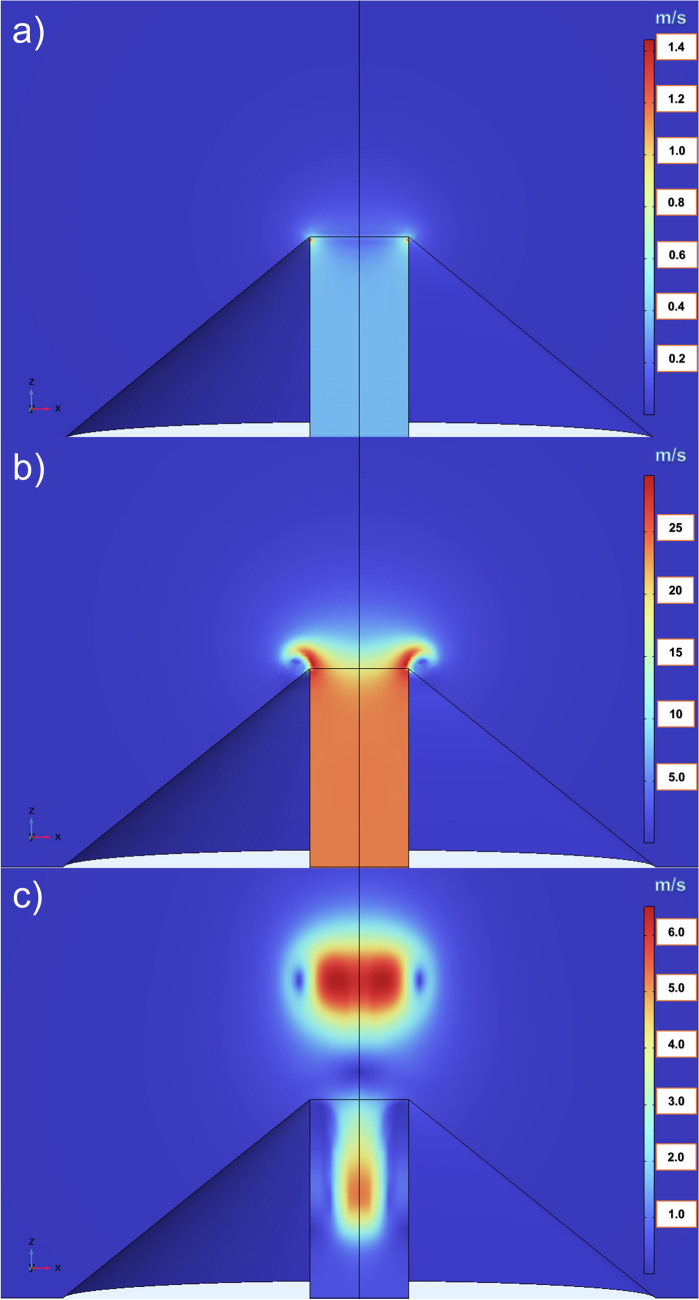
VVR formation visualized at (**a**) 0.01 s, (**b**) 0.1 s and (**c**) 1 s, after the starting of the simulation. The color scale represents the velocity in m/s.

Simulations show that, as an effect of the applied impulsive overpressure, the water vapor is pushed out of the puffer top, diffusing into the cold air domain. In particular, inside the puffer the fluid moves with constant velocity but at the puffer top it begins to curl up around the border, while the central part of the fluid moves up at higher velocity. A velocity gradient forms and causes the inner layers to roll around the outer layers forming a ring-shaped vortex. This process acts in tenths of a second, so the ring is emitted impulsively into the air domain. Due to its own momentum, thermal buoyancy and to the inertia of the rotating fluid, the vortex ring rises upwards in the atmosphere (Fig. 3). As the ring ascends, it slows and cools down due to diffusion and heat-transfer processes with the surrounding air.

### Overpressure and geometrical effects

**Figure 4:**
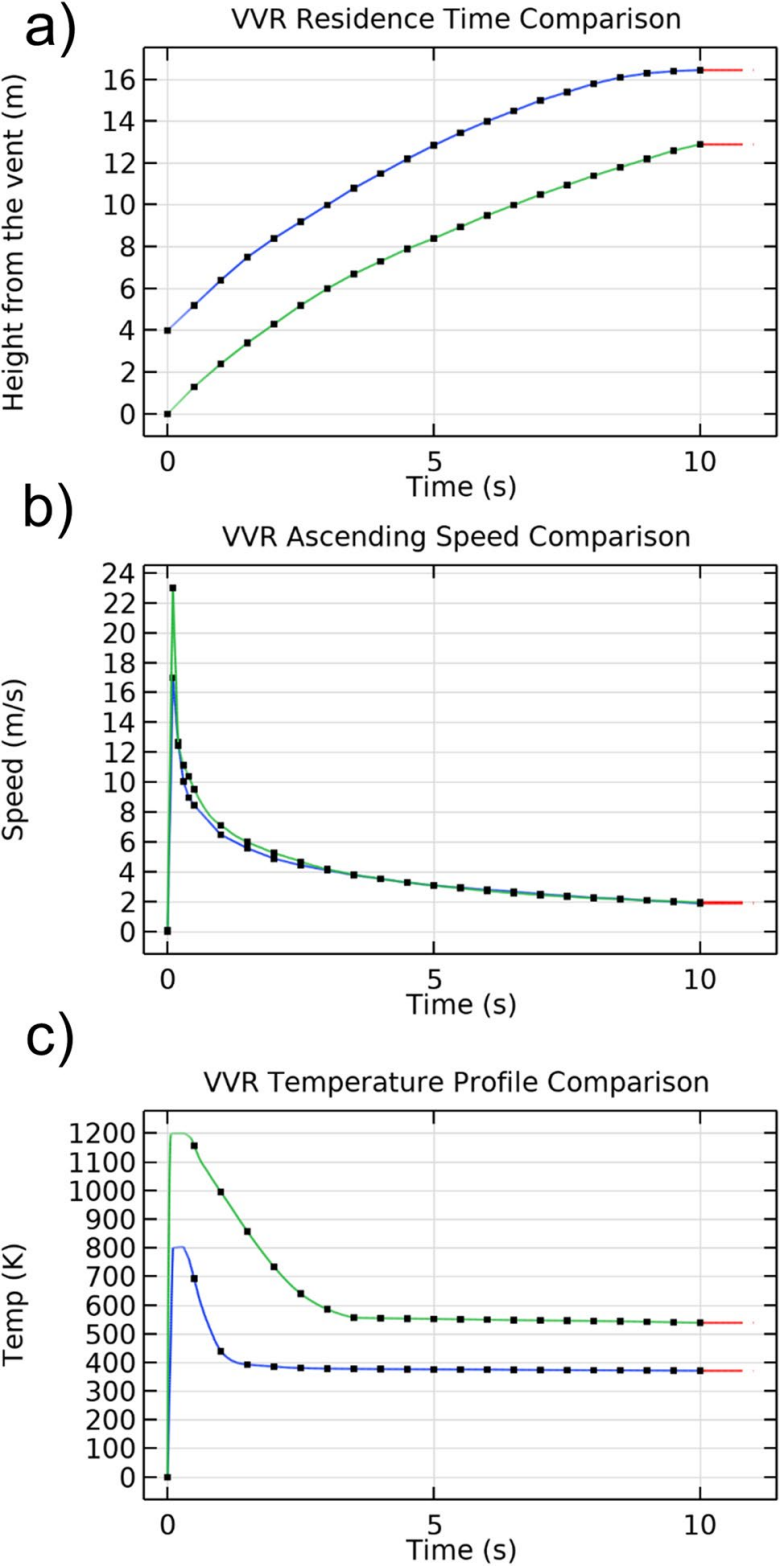
(**a**) Residence time (**b**) ascending speed and (**c**) temperature profiles of VVR obtained by modelling a puffer height of 4 m (blue line) and considering a flat-terrain case (green line). The red line represents a linear interpolation. The uncertainty is within the size of symbols.

We investigate the effect of the overpressure amplitude on the VVR generation. The conduit radius (d1) and the puffer top radius (d2) are set at 1 m while the puffer height (h1) is set at 4 m. We first perform three tests by applying 1 kPa, 10 kPa and 100 kPa max overpressure with a duration of 0.2 s. We believe this time to be reasonable for a small VVRs source like a puffer, considering that for bigger slugs (as the ones monitored with a Doppler radar on Erebus volcano by Gerst^44^ the recorded burst time is about 0.3–0.5 s. Results show that, for 1 kPa and 10 kPa the VVR does not form because viscous dissipation effects become predominant. At 100 kPa the VVR forms and has enough kinetic energy to leave the vent. VVR ascending speed, residence time and temperature profiles at 100 kPa are shown in Fig. 4. Speed and temperature profiles show respectively an instantaneous peak of about 17 m/s and an initial temperature of 800 K followed by exponential decay, as predicted by the theory^52^. After 10 s (end of simulation) VVR has travelled for about 12 m over the vent and has a temperature of 400 K. Additional tests applying intermediate overpressures show that the minimum overpressure to generate a ring is 40 kPa, that can explain why vortex rings cannot be seen during low intensity strombolian activity.

Concerning the geometrical effect, we first parametrize the puffer height (h1) to assume values of 3, 4, or 5 m and we check for differences in speed, temperature and residence time profiles. We find that differences are negligible. Moreover, we consider a flat-terrain case and we compare it to the case with h1 = 4 m. We observe that a flat terrain produces a ring with higher peak velocity and temperature. We think that differences in velocity and temperature are mainly the result of the reduced distance between the vent and the source of the pulse.

## Discussion

We investigated the formation of volcanic vortex rings (VVRs) by developing a 2D axi-symmetric time-dependent non-isothermal model, based on the finite element method. Simulations show that VVRs can be generated when water vapor is expelled from a circular vent of a small conical structure (puffer), due to the application of an impulsive overpressure, attributed to a gas slug explosion. The model input parameters (geometry of the puffer, slug overpressure and environmental conditions) were set according to field observations and literature. Simulated ring characteristics were compared to observations in terms of size, velocity, temperature, and residence time. Assuming the puffer as a truncated conical structure with a height of three to five meters and one-meter vent radius, our findings show that VVRs have a radius comparable to the vent and a small section diameter without trailing jets. Both characteristics are also observed in reality. Laboratory experiments performed by researchers (Supplementary Appendix A, Video 3) show that, for a circular nozzle, these characteristics occur only if the slug length (L) and the diameter of the emitting nozzle (D) are comparable (L/D ≤ 2). The ejection of a small quantity of gas, is in favor of a fast emitting source and confirms that the approach used for the simulations (fast overpressure pulse from a gas slug explosion) is valid. For the chosen geometry, this implies ejected gas volumes of 21–42 m^3^, which are consistent to independent findings by ^39,40^. We also find that geometrical aspects of the puffer are important to the VVR generation. For an applied overpressure of 100 kPa, velocity profiles taken along the symmetry axis, show peak velocities of about 17 m/s followed by exponential decay. We think that the peak value is reasonable since for bigger rings, like the ones observed in Sakurajima volcano^41^, estimated velocities are about 40 m/s. Additional simulations, using 500 kPa and 1000 kPa, over pressure showed that for 500 kPa, the ring still forms but the peak ring velocity reached 130 m/s which seems unlikely to occur on volcanoes. Instead for 1000 kPa we observed a jet. Those results are in agreement with results of the overpressure model proposed in Del Bello et al.^37^.

The shape of the velocity profile is consistent with analog experimental modeling of volcanic eruptions^53^, and with prevalent theory^52^. Results show also that the generation of VVRs is regulated by the balance between the momentum transferred to the water vapor and the thermal and mass diffusion processes. With the chosen geometry, the generation of a VVR requires a minimum overpressure of 40 kPa. Below this threshold, the VVR does not form because thermal and viscous diffusion processes become dominant. The existence of a minimum overpressure in natural volcanic systems can be explained as follow. Considering that the mafic melt at the free surface with the atmosphere would release the gas, each single bubble migrating through the melt, though pushed upwards by their extremely low density, must win a high resistance, and therefore the formation of volcanic vortex rings will be possible only if the gas within the bubble has a consistent overpressure proportional to the intrinsic viscosity of the melt. However, the melt/air free surface is not a common occurrence; more often gas released by the melt must pass through a plug of heavily fractured solidified rock as shown in Fig. 2. In this case the gas flux will pass through partly clogged and funnel shaped fractures which will maintain the gas pressure and velocity, thus allowing the gas to continue to have the minimal overpressure able to produce its nozzle velocity.

The model considers also thermal effects and our results show that VVRs initial temperature (at the vent) is comparable (or slightly lower) to the apparent magma temperature. As soon as the VVR gets far from the vent, its temperature decreases rapidly because of thermal exchange and adiabatic pressure equilibration with the surrounding cold air. This result is consistent with observations from thermal cameras, installed on Yasur volcano (Vanuatu). Finally, we are able to infer the VVR vertical distance profile from the vent (residence time). We find that after 10 s (assuming a perfect vertical motion and in absence of wind) the VVR has risen about 12 m from the vent. We are aware that a full representation of the dynamics of gas slug and of VVRs kinematics would require considering additional mechanisms (e.g., final speed of uprising gas slug, surface tension, magma shell porosity, water vapor phase change and external environmental conditions) but these would in turn require additional data, which are not available or directly measurable and more complex simulations (incorporating a conduit model) which would require a way higher computational cost. Since VVRs formation depend also on puffer geometrical characteristics, we speculate that a vorticity gradient occurs also when a part of the vent border is broken or if the vent border level is not uniform. In this case, the generated VVR may not form or could be subject to warping or temporary instabilities (wobbling). The possibility for the ring to overcome the warping and get back to a regular donut shape, or to be alternatively completely destroyed, depends on the initial warping level and on external environmental conditions. This may explain why VVRs don’t always form, and not on every volcano, but only under specific conditions.

Nevertheless, our model assumptions and corresponding results match several observed aspects and we consider our study an important first step for the investigation of such complex phenomena and for the understanding of the very shallow degassing processes in magma conduits. Future experimental data may shed more light on such a fascinating process.

## Methods

Following^50^ we use the following model that solves for momentum, energy and mass conservation equations.

M1 $$\rho \frac{\partial {\varvec{u}}}{\partial t}+\rho \left({\varvec{u}}\cdot \nabla \right){\varvec{u}}=\nabla \cdot \left[-p{\varvec{I}}+{\varvec{K}}\right]+{\varvec{F}}+\rho {\varvec{g}},$$

M2 $$\rho \nabla \cdot \left({\varvec{u}}\right)=0,$$

M3 $${\varvec{K}}=\left(\mu +{\mu }_{T}\right)\left(\nabla {\varvec{u}}+{\left(\nabla {\varvec{u}}\right)}^{T}\right),$$

M4 $$\rho \frac{\partial k}{\partial t}+\rho \left({\varvec{u}}\cdot \nabla \right)k=\nabla \cdot \left[\left(\mu +{\mu }_{T}{\sigma }_{k}\right)\nabla k\right]+{p}_{k}-{\beta }_{0}\rho \omega k,$$

M5 $$\rho \frac{\partial \omega }{\partial t}+\rho \left({\varvec{u}}\cdot \nabla \right)\omega =\nabla \cdot \left[\left(\mu +{\mu }_{T}{\sigma }_{\omega }\right)\nabla \omega \right]+\alpha \frac{\omega }{k}{p}_{k}-\rho {\beta }_{0}{\omega }^{2},$$

where

M6 $${\mu }_{T}=\rho \frac{k}{\omega },$$

M7 $${p}_{k}={\mu }_{T}\left[\nabla {\varvec{u}}:\left(\nabla {\varvec{u}}+{\left(\nabla {\varvec{u}}\right)}^{T}\right)\right].$$

Equations (M1)–(M7) represent respectively the conservation of momentum, the conservation of mass, the viscous stress tensor, the transport equation for the turbulent kinetic energy (K), the transport equation for the specific dissipation rate (ω), the turbulent viscosity and the production term. Where P is the pressure, I is the identity tensor, F is the vector of the external forces applied to the fluid, g is the gravity acceleration vector, and ${\sigma }_{k}$, ${\sigma }_{\omega }$, ${\beta }_{0}$, $\alpha$ are turbulent model parameters. Streamline stabilization is applied to help fulfill the Babuska–Brezzi condition^54^. Because our model is non-isothermal, we also solve for the temperature variation with the heat transfer equation:

M9 $$\rho {C}_{p}\frac{\partial T}{\partial t}+\rho {C}_{p}{\varvec{u}}\cdot \nabla T+\nabla \cdot {\varvec{q}}=Q+{Q}_{p}+{Q}_{vd},$$

where ρ is the density, Cp is the heat capacity at constant pressure, **u** is the velocity vector, T is the temperature, **q** is the heat flux defined as:

M10 $${\varvec{q}}=-k\nabla T,$$

with k the thermal conductivity, Q contains the heat sources other than viscous dissipation, while Q_p_ and Q_vd_ are defined as:

M11 $${Q}_{p}={\alpha }_{p}T\left(\frac{\partial p}{\partial t}+{\varvec{u}}\cdot \nabla p\right),$$

and

M12 $${Q}_{vd}=\tau :\nabla {\varvec{u}},$$

(with α_p_ the coefficient of thermal expansion and τ the viscous stress tensor) and represent the work done by pressure changes and the viscous dissipation in the fluid respectively.

The fluid dynamic viscosity dependence from the temperature is taken into consideration through the Sutherland equation^55^:

M13 $$\mu ={\mu }_{ref}{\left(\frac{T}{{T}_{ref}}\right)}^\frac{3}{2}\frac{{T}_{ref}+{S}_{\mu }}{T+{S}_{\mu }},$$

where µ is the dynamic viscosity, µ_ref_ the reference dynamic viscosity taken as 1.12e^−5^ for water vapor and 1.72e^−5^ for air, T the temperature, varying between 1300 and 273 K, ${T}_{ref}$ is the reference temperature and ${S}_{\mu }$ the Sutherland coefficient that is 1064 for water vapor and 111 for air.

Mass transfer and diffusion processes in air domain are taken into consideration treating the water vapor as a diluted specie and solving the convection–diffusion equation:

M12 $$\frac{\partial c}{\partial t}+\nabla \cdot \left(c{\varvec{u}}\right)=\nabla \cdot \left(\mathrm{D}{\nabla }_{c}\right)+R,$$

where c is the concentration (whose initial values are 1 for the water vapor and 0 for air), D is the diffusivity, R describes source or sinks of the variable (the chemical specie) c.

## Supplementary Information

Supplementary Information.
