# Supplementary material for: Dynamics of volcanic vortex rings
Source: Sci Rep. 2023 Feb 9;13:2369. doi: 10.1038/s41598-022-26435-0 (PMC9911723; doi:10.1038/s41598-022-26435-0)
Supplement: Supplementary file 1 — Supplementary Information. [file 41598_2022_26435_MOESM1_ESM.docx]

**Appendix A - Supplementary videos free available at the publication date**

Video 1 (Rarely seen smoke rings at Mt Etna)

<https://www.youtube.com/watch?v=qeJ-09MEj00&fbclid=IwAR0cY-DTVqhpfHWPfwhoMariquPTEagUi_HJUgyljRCvBCzd1jxlMf9u5SYk>

Video 2 (Vortex ring observed at Momotombo)

<https://www.youtube.com/watch?v=sNIKDrUO29Y>

Video 3 (Evolution and Breakdown of Elliptic Vortex Ring)- (Adhikari and Lim)

<https://www.youtube.com/watch?v=oGGRxE2ijl0>

**Appendix B - Articles**

Article 1 (National Geographic Interview and rings on thermal cameras)

<https://www.nationalgeographic.com/science/article/volcanoes-blow-smoke-rings-now-we-know-how>

Article 2 (Just an article on BBC news)

<http://news.bbc.co.uk/2/hi/science/nature/696953.stm>

Article 3 (Pictures of rings from Dec 2002 Stromboli eruption, Copyright: M. Fulle).

<https://www.swisseduc.ch/stromboli/volcano/sciara0203/preflow-en.html>

Article 4 (Ring from Pacaya volcano in 2005)

<https://skagwaydelta.wordpress.com/2011/05/15/pacaya-volcano-blows-a-smoke-ring-in-farewell-guatemala-2005/>

**Appendix C - Supplementary photos**

Photos 1 (Pictures from Stromboli eruption June 2006)

<https://www.volcanodiscovery.com/fr/photos/stromboli/0606/smokerings.html>

Photos 2 (Pictures of rings from Etna, Stromboli and Tungurahua)

<http://www.earth-of-fire.com/article-les-volcans-fument-la-pipe-formation-de-vortex-toroidal-69760899.html>

Photos 3 (Picture of ring from Mt. Redoubt-Alaska)

<https://www.flickr.com/photos/31220278@N05/3880927815/>

Photos 4 (A picture of ring from Aso volcano)

<https://www.flickr.com/photos/mikelyvers/6890803977/>

Photos 5 (Pictures of rings at white island)

https://www.flickr.com/photos/mr_step/albums/72157632870078288

**Appendix D**

**
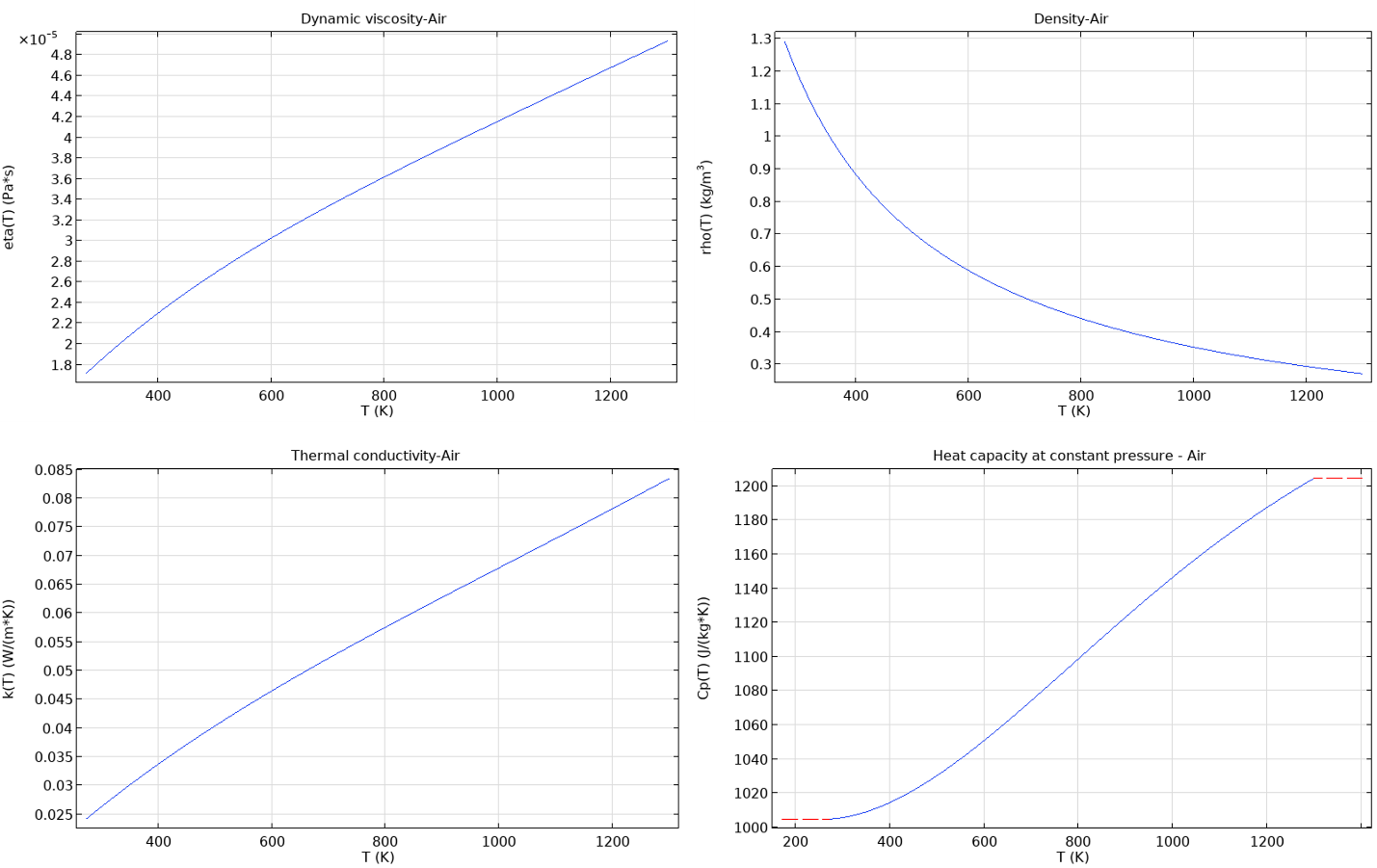
**

**
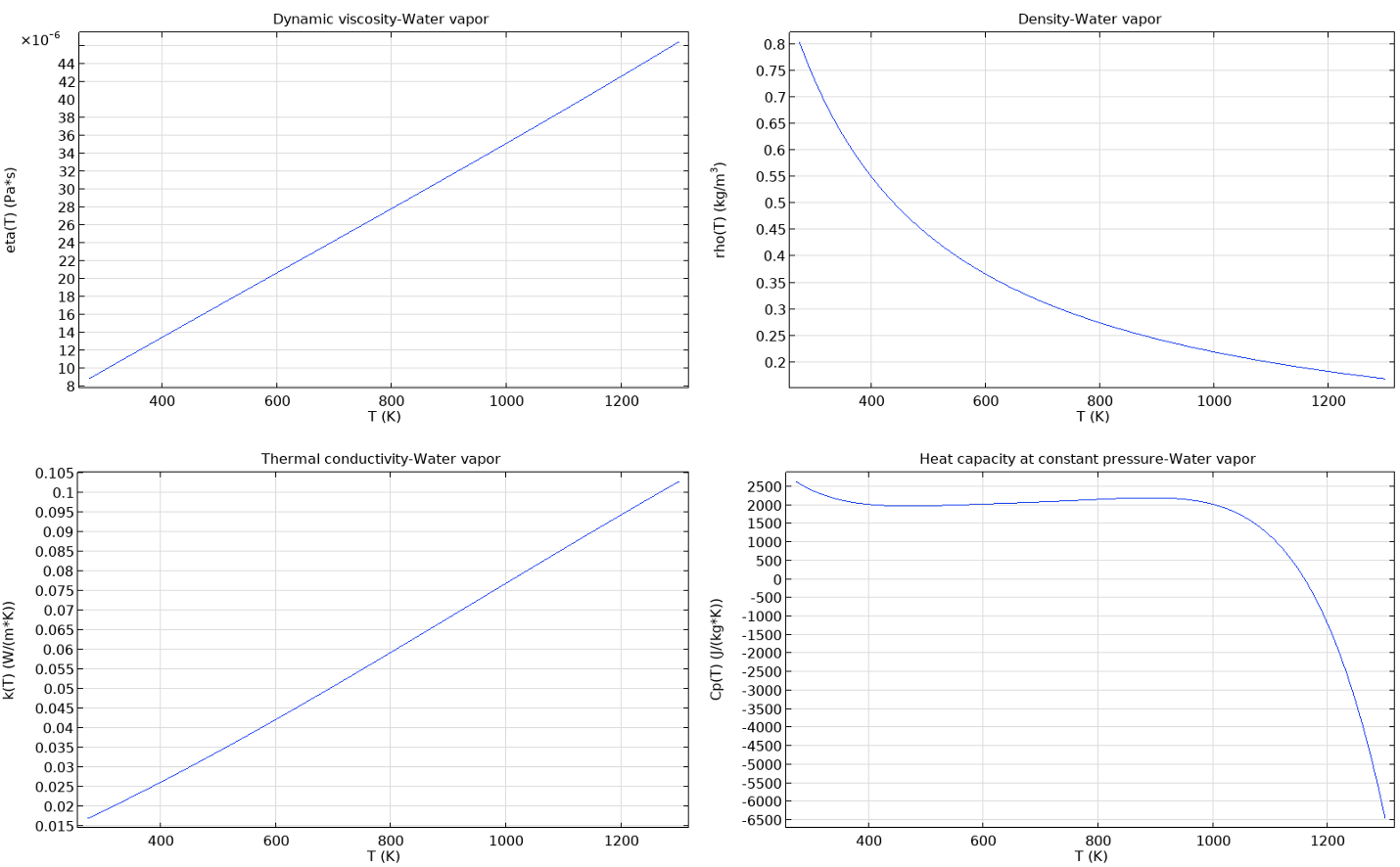
**

**Figure D1.** Dynamic viscosity, density, thermal conductivity and heat capacity at constant pressure as function of temperature in the range 270-1300K for air and water vapor.
